# Mindful breathing as an effective technique in the management of hypertension

**DOI:** 10.3389/fphys.2023.1339873

**Published:** 2024-01-23

**Authors:** Aravind Natarajan, Hulya Emir-Farinas, Hao-Wei Su

**Affiliations:** Google LLC, Mountain View, CA, United States

**Keywords:** hypertension, mindful breathing, meditation, blood pressure, non-pharmacological methods

## Abstract

**Introduction:** Hypertension is one of the most important, modifiable risk factors for cardiovascular disease. The popularity of wearable devices provides an opportunity to test whether device guided slow mindful breathing may serve as a non-pharmacological treatment in the management of hypertension.

**Methods:** Fitbit Versa-3 and Sense devices were used for this study. In addition, participants were required to own an FDA or Health Canada approved blood pressure measuring device. Advertisements were shown to 655,910 Fitbit users, of which 7,365 individuals expressed interest and filled out the initial survey. A total of 1,918 participants entered their blood pressure readings on at least 1 day and were considered enrolled in the study. Participants were instructed to download a guided mindful breathing app on their smartwatch device, and to engage with the app once a day prior to sleep. Participants measured their systolic and diastolic blood pressure prior to starting each mindful breathing session, and again after completion. All measurements were self reported. Participants were located in the United States or Canada.

**Results:** Values of systolic and diastolic blood pressure were reduced following mindful breathing. There was also a decrease in resting systolic and diastolic measurements when measured over several days. For participants with a systolic pressure ≥ 130 mmHg, there was a decrease of 9.7 mmHg following 15 min of mindful breathing at 6 breaths per minute. When measured over several days, the resting systolic pressure decreased by an average of 4.3 mmHg.

**Discussion:** Mindful breathing for 15 min a day, at a rate of 6 breaths per minute is effective in lowering blood pressure, and has both an immediate, and a short term effect (over several days). This large scale study demonstrates that device guided mindful breathing with a consumer wearable for 15 min a day is effective in lowering blood pressure, and a helpful complement to the standard of care.

## 1 Introduction

Hypertension, or high blood pressure is a very common condition, affecting an estimated 44.4% of adults in the United States (Ritchey et al., 2018). The 2017 guideline for high blood pressure in adults (Whelton and Carey, 2018) defines elevated blood pressure as having a systolic pressure in the range 120–129 mmHg and a diastolic pressure <80 mmHg. Stage 1 hypertension is defined as having a systolic pressure in the range 130–139 mmHg or a diastolic pressure in the range 80–89 mmHg. Stage 2 hypertension is defined as having a systolic pressure ≥ 140 mmHg or a diastolic pressure ≥ 90 mmHg. Hypertension is associated with major health risks (Nguyen et al., 2010; Buford, 2016; Fisher and Curfman, 2018; Kjeldsen, 2018), and is a component of cardiovascular risk scores (D Agostino et al., 2008), as well as metabolic syndrome severity scores (Wiley and Carrington, 2016). Only about half of all adults with hypertension had their blood pressure controlled in 2015–2016 (Fryar et al., 2017).

Autonomic imbalance, i.e., sympathetic overactivity and/or parasympathetic underactivity plays a pivotal role in the etiology of essential hypertension (Brook and Julius, 2000). Chronic stress has been associated with the development of hypertension through sustained blood pressure elevations over time (Spruill, 2010). Among lifestyle choices that help to mitigate hypertension is the practice of mindful breathing (Joseph et al., 2005; Kaushik et al., 2006; Tyagi and Cohen, 2014; Cernes and Zimlichman, 2017; Li et al., 2018; Brenner et al., 2020). Slow breathing, e.g., at 6 breaths per minute is an effective technique in preserving autonomic function, resulting in high respiratory sinus arrhythmia, and baroreflex sensitivity (Russo et al., 2017). In hypertensive patients, breathing at a rate of 6 per minute has been shown to increase baroreflex sensitivity to values similar to those of healthy controls who were breathing spontaneously (Joseph et al., 2005). Several studies have investigated the effect of slow, mindful breathing on hypertension and the results have been mixed: some studies involving device guided mindful breathing have found evidence for the reduction in blood pressure due to mindful breathing (Grossman et al., 2001; Rosenthal et al., 2001; Schein et al., 2001; Viskoper et al., 2003; Elliot et al., 2004; Joseph et al., 2005; Elliott and Izzo, 2006; Brook et al., 2013; Howorka et al., 2013; Chaddha et al., 2019) while other studies have shown no benefit (Altena et al., 2009; Landman et al., 2014; Mahtani et al., 2016).

Guided breathing devices already exist, for example, Resperate^1^ is an FDA-cleared commercial product designed to lower blood pressure using melody-guided breathing. A systematic review and meta-analysis of 8 trials of the Resperate device with trial arms of 4 weeks or greater, was conducted by Mahtani, Nunan, and Heneghan (Mahtani et al., 2012). Their analysis found that overall device guided breathing resulted in a decrease of systolic pressure by 3.06 mmHg and diastolic pressure by 2.35 mmHg. However when studies that were sponsored by or involved the manufactures of the device were excluded, there was no significant overall effect in either systolic or diastolic blood pressure. Thus, there is a need for further research on this topic. The rise in the popularity of wearable devices such as smartwatches, trackers, and rings ensures that research on mindful breathing can benefit a large number of people.

In this article, we investigate the effect of device guided slow mindful breathing on blood pressure, using Fitbit devices. We show that 15 min of mindful breathing at a frequency of six breaths per minute is effective in temporarily lowering both systolic and diastolic blood pressure. Furthermore, the practice of mindful breathing over time is shown to lower the resting blood pressure.

## 2 Methods

Participants were enrolled through a recruitment banner shown on the Fitbit App running on their mobile device. We targeted Fitbit users 21 years of age or older, and who resided in the United States or Canada. Participants were required to own a blood pressure device approved by the FDA or Health Canada. Among the exclusion criteria were participants who were pregnant at the time of the study, participants who were diagnosed with heart arrhythmias, or regular users of tobacco products based on self report. All participants owned either a Fitbit Sense or a Fitbit Versa-3 device. Participants were enrolled between 1 June 2022 and 31 July 2022, and the study lasted 28 days. All study materials were approved by an Institutional Review Board (Advarra IRB).

The recruitment banner was shown to 655,910 Fitbit users, and informed consent was obtained electronically. Participants were provided with instructions to download the HYPERTENSION app onto their Fitbit device. A second app (henceforth called the “Program”) running on their mobile device (within the Fitbit app) was used to collect data from participants.

The HYPERTENSION app running on the Fitbit device provided a guided breathing experience. The app consisted of an expanding and contracting circle meant to guide inhalation and exhalation. The guide frequency was set to six breaths per minute (5 s inhalation, 5 s exhalation), and the session length was set to 15 min. The same protocol was applied to both male and female participants since the increase in baroreflex sensitivity due to slow, mindful breathing is expected to be independent of sex (Adler et al., 2019; Shafiq et al., 2023).

Participants were encouraged to engage with the HYPERTENSION app once per day, prior to sleep. Before starting the app, participants took a blood pressure reading using their own blood pressure measurement device, and entered the systolic and diastolic values into the Program on their mobile device. They then engaged with guided breathing using the HYPERTENSION app. Upon completing the guided breathing session, participants took their blood pressure again and entered the values into the Program. Timestamps were used to validate data: we only retained data if the blood pressure values were entered into the app sooner than 20 min following completion of the mindful breathing session. We excluded data that had systolic and diastolic entries that were the same as the default values (systolic pressure = 120 mmHg and diastolic pressure = 80 mmHg). No materials were mailed to participants, and no remuneration was made for participation. There were a total of 11,791 measurements of blood pressure, and 97.1% of measurements were made between 6 p.m. and 6 a.m.

Participants were surveyed as to their race and ethnicity, hypertension status, medication usage, and prior meditation experience. 7,365 Fitbit users expressed interest in the program. Of the 7,365 potential participants who filled out the survey, 1,918 participants entered their blood pressure on at least 1 day. Five hundred and ninety seven participants entered blood pressure data on 7 days or more, 307 participants had 14 or more days of data, 115 had 21 or more days of data, and 2 participants entered data for all 28 days. Among the 1918 participants who provided blood pressure data on at least 1 day, 76.4% identified as White or Caucasian, 10% as Black or African-American, 4.6% as Hispanic or Latino, 2.6% as Asian, 0.9% as American Indian or Alaska Native, 0.1% as Native Hawaiian or other Pacific Islander. The rest identified as multi-racial, or declined to provide information. 17.3% indicated they had prior meditation/mindfulness experience, 82.5% said they did not have any meditation/mindfulness experience, and 0.2% did not answer. 47.4% indicated that they were taking medication for hypertension, 14% were not taking medications to manage their hypertension, 37.7% were not diagnosed with hypertension, and 0.8% did not respond to the question.

**FIGURE 1:**
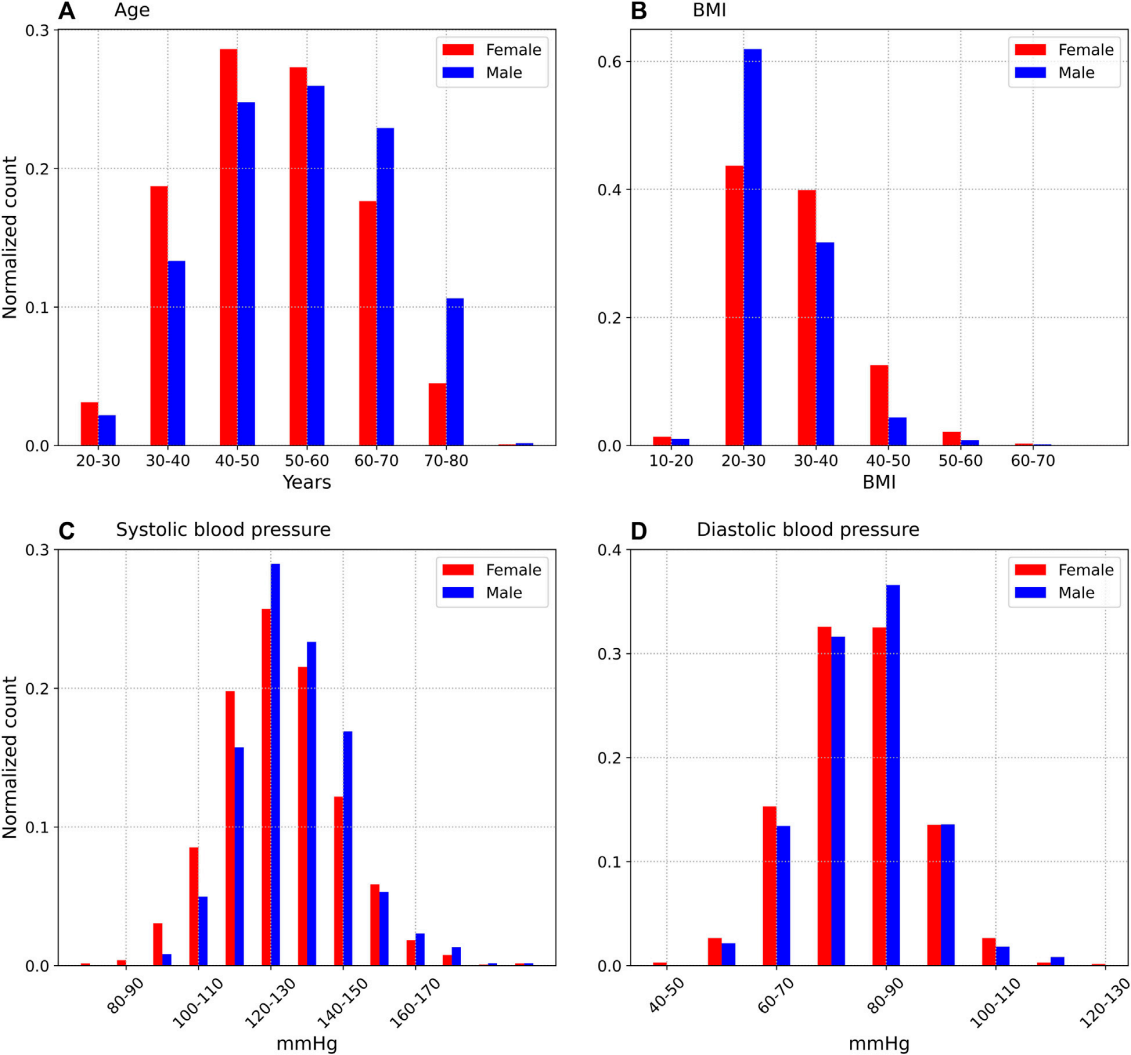
Age **(A)**, BMI **(B)**, systolic **(C)**, and diastolic **(D)** distributions of subjects, on the first day of the study. Only the participants who provided 1 or more days of blood pressure data are included.

Figure 1A shows the age distribution for female and male participants. The median age was 54 years (the interquartile range (IQR) was 43–62 years) for males, and 49 years (IQR 41–58 years) for females. Females accounted for 68.9% of participants. Figure 1B shows the distribution of body mass index (BMI). The median BMI was 28.0 (IQR 25.5–32.1) for males, and 31.0 (IQR 26.3–36.9) for females. Figures 1C, D show the distribution of the first measurement of systolic and diastolic pressures respectively. The median systolic pressure for the first measurement was 129 mmHg (IQR 121–140 mmHg) for males, and 127 mmHg (IQR 116–137 mmHg) for females. The median diastolic pressure was 80 mmHg (IQR 73.75–87.0 mmHg) for males and 79.0 mmHg (IQR 72.0–86.0 mmHg) for females.

For the analysis of blood pressure decrease over several days, we included a comparison with a control set. The control set consisted of 51 individuals with 7 or more days of data, and was extracted from a different study that ran between April and June 2021. Blood pressure readings for the control were self reported in the mornings, and the participants were not instructed to practice mindful breathing. The average age of the participants in the control set was 58.8 years, and 64.7% were male. Although the control set had a higher proportion of males than the treatment set, we do not think that the decrease in blood pressure due to mindful breathing is dependent on sex (Adler et al., 2019; Shafiq et al., 2023). Among the control set, 39.2% reported not having been diagnosed with hypertension, while 49% indicated they were taking hypertension medication. Control data were collected under the guidance of an Institutional Review Board (Advarra IRB).

## 3 Results

In this section, we will discuss how device guided mindful breathing is associated with: i) an immediate effect, i.e., decrease in blood pressure following the practice, and ii) a medium term effect, i.e., decrease in blood pressure over several days.

### 3.1 Decrease in blood pressure immediately following mindful breathing

Participants measured their systolic and diastolic blood pressure both before (initial reading) and after (final reading) each 15 min mindful breathing session. Since blood pressure can be increased by ambulatory movement, it is important to ensure that participants were at rest prior to starting the mindful breathing session. Infrared photoplethysmography data was used to detect when the device is being worn. We enforced the condition that the device is worn during the 5 min period prior to starting the mindful breathing protocol. We further require that the number of steps taken in that 5 min period is less than 10. Enforcing these two conditions reduces the number of measurements from 11,746 to 3,867. We describe the effect of steps on blood pressure in more detail in the Supplemental Material.

**FIGURE 2:**
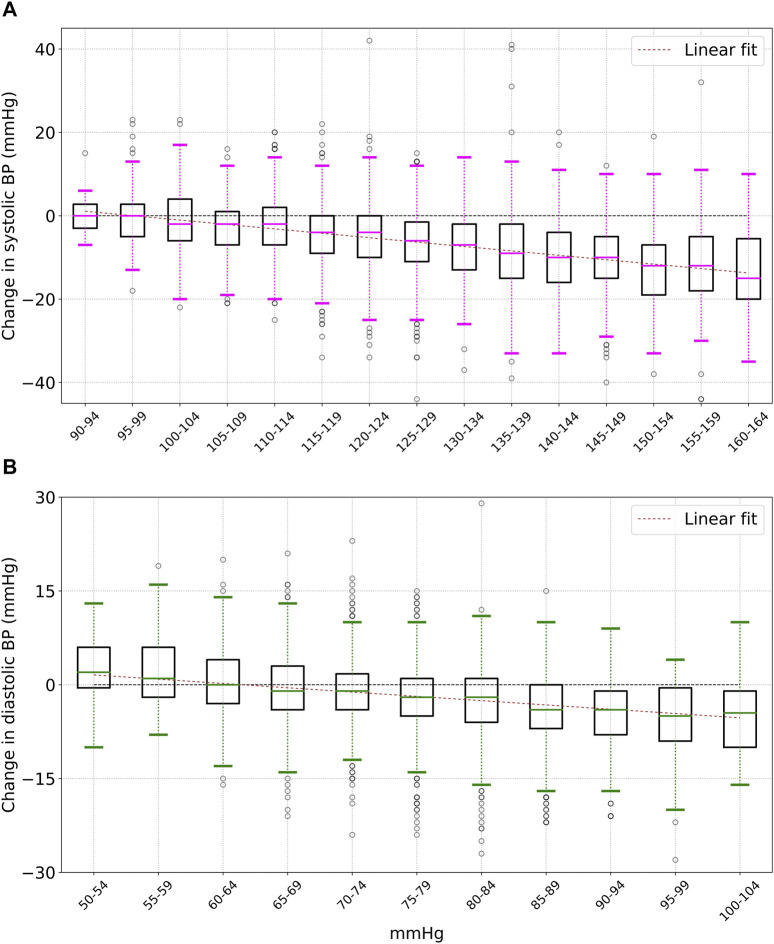
Change (after - before) in systolic **(A)** and diastolic **(B)** blood pressure after 15 min of mindful breathing. There is an approximately linear dependence on the initial blood pressure. The boxes show the interquartile range (IQR), the horizontal bars show the median, and the whiskers are 1.5 times the IQR.

Figure 2 shows the difference between the readings: Δ = (final reading−initial reading), for various values of initial systolic and diastolic blood pressure within a bin size of 5 mmHg. We only consider bins with at least 25 measurements. The box represents the interquartile range (IQR) and the median values are shown by horizontal bars. The whiskers represent limits that are 1.5 times the IQR. The points above and below the whiskers are outliers. Also shown is a linear fit to the median values:

$$\Delta_{fit\;}=\Delta_{0}\;\left(1-\frac{\alpha }{\alpha_{0}}\;\right)$$ (1)

where Δ_fit_ is a linear fit that represents the change in blood pressure (systolic or diastolic), and α is the blood pressure (systolic or diastolic) measured prior to the mindful breathing intervention. The constants (Δ_0_, α_0_) in Eq. 1 are (20.5, 97.0) mmHg for systolic blood pressure, and (8.7, 63.4) mmHg for diastolic blood pressure. The fit implies that blood pressure is decreased when the initial systolic pressure >97 mmHg or the diastolic pressure >63.4 mmHg. The reduction in blood pressure is larger for larger initial measurements. For values of systolic (diastolic) pressure >> α_0_, the estimated decrease in systolic (diastolic) blood pressure are estimated to be 20.5 (8.7) mmHg respectively.

The median change in systolic and diastolic blood pressure following mindful breathing, for various values of initial blood pressure are shown in Table 1. For participants with an initial systolic pressure ≥ 130 mmHg, the mean (standard deviation) for the change in systolic pressure was −9.7 (10.2) mmHg. For diastolic pressure, the corresponding change was −3.9 (6.4) mmHg. Similar results were obtained for males and females—For male participants with an initial systolic ≥ 130 mmHg, the mean (standard deviation) for the decrease in systolic pressure was −9.4 (9.9) mmHg, while for female participants, the values were −9.8 (10.3) mmHg (the difference is not statistically significant). Similar results were also obtained for older and younger participants—For participants of age ≥ 50 years (as of 1 June 2022) with an initial systolic measurement ≥130 mmHg, the mean (standard deviation) for the decrease in systolic was found to be −9.9 (10.3) mmHg. For participants younger than 50, the corresponding values were −9.3 (9.9) mmHg (the difference is not statistically significant).

**TABLE 1:** Median change in systolic and diastolic blood pressure after completing a 15 min mindful breathing session. The interquartile range (IQR) is the 25th to 75th percentile range. The change is strongly dependent on initial blood pressure.

Systolic (mmHg)	Median change in systolic (mmHg)	IQR (mmHg)	Diastolic (mmHg)	Median change in diastolic (mmHg)	IQR (mmHg)
90–94	0.0	(−3.0, 2.8)	50–54	2.0	(−0.5, 6.0)
95–99	0.0	(−5.0, 2.8)	55–59	1.0	(−2.0, 6.0)
100–104	−2.0	(−6.0, 4.0)	60–64	0.0	(−3.0, 4.0)
105–109	−2.0	(−7.0, 1.0)	65–69	−1.0	(−4.0, 3.0)
110–114	−2.0	(−7.0, 2.0)	70–74	−1.0	(−4.0, 1.8)
115–119	−4.0	(−9.0, 0.0)	75–79	−2.0	(−5.0, 1.0)
120–124	−4.0	(−10.0, 0.0)	80–84	−2.0	(−6.0, 1.0)
125–129	−6.0	(−11.0, −1.5)	85–89	−4.0	(−7.0, 0.0)
130–134	−7.0	(−13.0, −2.0)	90–94	−4.0	(−8.0, −1.0)
135–139	−9.0	(−15.0, −2.0)	95–99	−5.0	(−9.0, −0.5)
140–144	−10.0	(−16.0, −4.0)	100–104	−4.5	(−10.0, −1.0)
145–149	−10.0	(−15.0, −5.0)			
150–154	−12.0	(−19.0, −7.0)			
155–159	−12.0	(−18.0, −5.0)			
160–164	−15.0	(−20.0, −5.5)			

**FIGURE 3:**
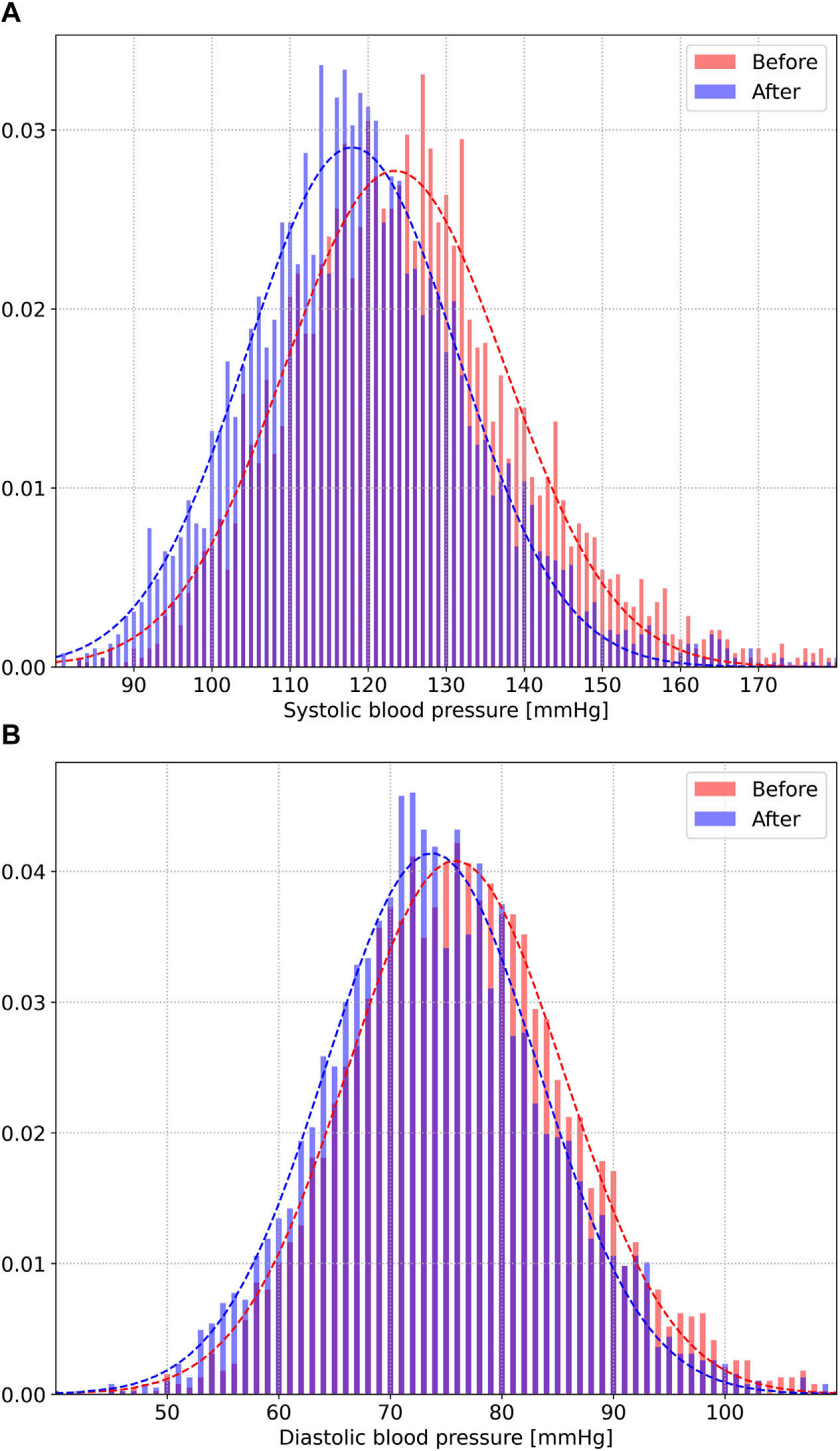
Distributions of systolic **(A)** and diastolic **(B)** blood pressure before (red) and after (blue) 15 min of mindful breathing.

Figure 3 shows the distributions of systolic (a) and diastolic (b) blood pressure before (red) and after (blue) mindful breathing. The mean (standard deviation) of all systolic blood pressure values prior to starting the mindful breathing practice was 125.3 (15.6) mmHg, with 3,867 measurements. The mean (standard deviation) of all systolic measurements after completing mindful breathing was 119.3 (14.9) mmHg, with a Cohen-d effect size of 0.3925 (*p* < 0.0001). For diastolic pressure, the mean (standard deviation) of all measurements was 76.5 (10.1) mmHg. The mean (standard deviation) following mindful breathing was 74.4 (10.1) mmHg, with a Cohen-d effect size of 0.2144 (*p* < 0.0001).

### 3.2 Medium term effect on resting blood pressure

**FIGURE 4:**
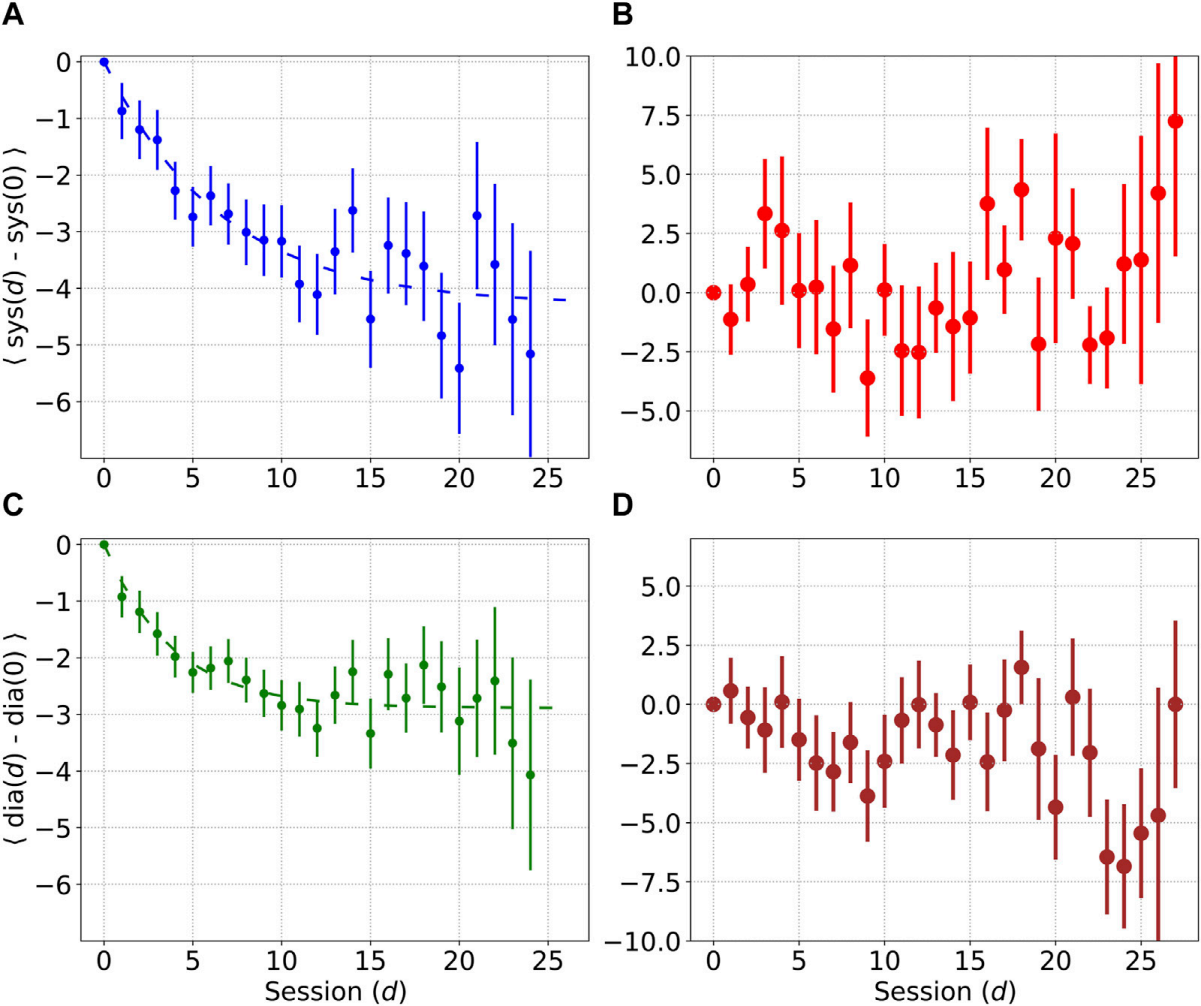
Mean decrease in systolic and diastolic blood pressure d days after the start of the practice. Error bars denote the standard error of the mean. Plots **(A)** and **(C)** show decrease in blood pressure in the treatment set, while plots **(B)** and **(D)** show decrease in blood pressure in the control set.

Let us now consider the medium term impact of mindful breathing on blood pressure. Figure 4 shows the medium term decrease in systolic and diastolic blood pressure *d* days after starting the mindful breathing practice. Let *S*_*i*_ *(0)* and *D*_*i*_ *(0)* be the systolic and diastolic blood pressure measurements on the first day of the experiment, for participant *i*. Similarly, let *S*_*i*_ *(d)* and *D*_*i*_ *(d)* be the systolic and diastolic measurements on day *d*. All measurements are made prior to mindful breathing. Let < *S(d)−S(0) >* and < *D(d)−D(0) >* represent the difference in systolic and diastolic measurements compared to the values prior to the start of the practice, i.e., on day 1, and averaged over all individuals. The error bars in Figure 4 represent the standard error of the mean.

The dotted lines indicate the following fitting function:

$$\Delta_{\text{rest}}=K\;\left[1-e^{-\left(d/d_{*}\right)}\right]$$ (2)

where Δ_rest_ may be < *S(d)−S(0) >* for systolic pressure, or < *D(d)−D(0) >* for diastolic pressure, and quantifies the reduction in resting blood pressure. d_*_ is a constant, and d = 0 represents the first day. For systolic measurements, we find the best fit values K = −4.3 mmHg and d_*_ = 6.6 days, while for diastolic measurements, the best fit values were found to be K = −2.9 mmHg, and d_*_ = 3.8 days. The time required for the systolic and diastolic measurements to fall to 90% of their asymptotic values was found to be 15.2 days and 8.8 days respectively.

We also performed this analysis separately for different groups based on hypertension status, and medication usage. Among participants diagnosed with hypertension without medication, the best fit value for K for the decrease in systolic pressure was found to be −4.9 mmHg (sample size = 87). Among participants diagnosed with hypertension who were being treated, the best fit for systolic pressure was K = −4.5 mmHg (sample size = 308). Among participants not diagnosed with hypertension, the best fit for systolic pressure was K = −2.8 mmHg (sample size = 198). Thus, the best long term results were found in participants with untreated hypertension, although we acknowledge that the sample sizes were small, especially for the cohort with untreated hypertension.

Equation 2 implies a dose-dependent relationship (we define “dose” as a 15-min mindful breathing session) between the reduction in resting blood pressure and length of the practice for d << d_*_, i.e., Δ_rest_ is proportional to the number of days, for small values of *d*. To quantify this relationship, we considered the data for the first 7 days, i.e., from *d* = 1 to *d* = 7, and fitted the data using two fitting functions: i) a linear model parameterized by a slope and an intercept, i.e., *k* = 2 variables, and ii) a constant parameterized by *k = 1* variable, and computed the chi-squared and likelihood functions:

$$\chi^{2}\left(k\right)={\left(\frac{y_{true}\;-\;y_{fit}}{\sigma }\right)}^{2}$$

$$L\left(k\right)\propto \exp-\frac{1}{2}\chi^{2}\left(k\right)$$ (3)

In Eq. 3, *y*_*true*_ represents the values of Δ_rest_, *y*_*fit*_ represents the fitting function, and σ is the standard error. We find $\chi^{2}$ (k = 2) = 1.867 with five degrees of freedom, and $\chi^{2}$ (k = 1) = 13.123 with 6 degrees of freedom. The likelihood ratio test:

$$\lambda =-2\;\ln\;\left(\frac{L\;\left(k=1\right)}{L\;\left(k=2\right)}\right)$$

$$=\chi^{2}\left(k=1\right)\;-\;\chi^{2}\left(k=2\right)$$ (4)

gives λ = 11.256. For a chi-squared distribution with 1 degree of freedom, this value of λ implies a *p-*value of 0.00079. A similar computation for the diastolic pressure, gives a *p-*value of 0.00189. There is thus, strong statistical evidence in favor of a dose-dependent relationship between reduction in blood pressure and number of days for small *d*. For *d* >> d_*_, the resting blood pressure is in the saturation region, and approaches a constant value.

Let us compare the treatment group and control group in the saturation region from d = 8 to d = 21 (14 days). For the treatment group, the difference between a linear fit and a constant is not statistically significant in this regime, for either the change in systolic pressure (*p* = 0.1444), or diastolic pressure (*p* = 1). We may thus model the blood pressure in this regime by a constant. Let the null hypothesis be that there is no effect, and let us compare the constant fit (Δ_rest_ = constant) to the null hypothesis (Δ_rest_ = 0). For the constant fit, we find $\chi^{2}$ (k = 1) = 10.383 for the systolic pressure, and $\chi^{2}$ (k = 1) = 5.171 for the diastolic pressure, with 13 degrees of freedom. Similar calculations for the null hypothesis yield $\chi^{2}$ (k = 0) = 291.3 for the systolic pressure and $\chi^{2}$ (k = 0) = 338.0 for the diastolic pressure, with 14 degrees of freedom. Applying the likelihood ratio test (Eq. 4), we may exclude the null hypothesis at *p* < 0.0001.

Now let us perform the same calculation for the control data. In the same 14 days region (from d = 8 to d = 21), the control data for the systolic measurements fitted to a constant Δ_control,sys_ = 0.0624 yields $\chi^{2}$ (k = 1) = 11.830 with 13 degrees of freedom. The null hypothesis for the systolic pressure gives us $\chi^{2}$ (k = 0) = 11.854 with 14 degrees of freedom. The difference is not statistically significant, and we conclude that the null hypothesis Δ_control,sys_ = 0 is acceptable. For the diastolic pressure, fitting the data with a constant Δ_control,dia_ = −1.3 gives us $\chi^{2}$ (k = 1) = 10.088 with 13 degrees of freedom. The null hypothesis for the diastolic pressure gives us $\chi^{2}$ (k = 0) = 14.924 with 14 degrees of freedom. The likelihood ratio test gives us a difference λ = $\chi^{2}$ (k = 0) - $\chi^{2}$ (k = 1) = 4.8355 implying *p* = 0.0279 for a chi squared distribution with 1 degree of freedom, excluding the null hypothesis at >97% confidence. There is thus weak evidence that unlike the systolic pressure, the control set shows a slight decrease in diastolic pressure. The results are summarized in Table 2.

**TABLE 2:** Decrease in resting and diastolic measurements taken over several days. When accounting for the control set, we see an average decrease of 4.3 mmHg in systolic pressure, and 1.6 mmHg in diastolic pressure.

	Systolic	Diastolic
Asymptotic value of Δ_rest_ (mmHg)	−4.3	−2.9
Days to reach 90% of asymptotic value	15.2	8.7
Estimated Δ_control_ from the control set measurements (mmHg)	0^a^	−1.3
Estimated reduction due to mindful breathing (mmHg)	−4.3	−1.6
Days to reach 90% of asymptotic value	15.2	8.8

^a^ Consistent with the null hypothesis.

## 4 Discussion

This study, the largest of its kind, shows that device guided breathing lowers blood pressure in the immediate and short term. Slow breathing at a frequency of six breaths per minute has been shown to improve alveolar ventilation and increase heart rate variability and baroreflex sensitivity (Russo et al., 2017). The frequency of 6 breaths per minute, or 0.1 Hz is similar to the frequency of Mayer waves, i.e., oscillations of arterial pressure (Julien, 2006; Julien, 2020). Thus breathing at this frequency can lead to the synchronization of respiration, heart rate, and blood pressure, leading to the condition of resonance, or coherence (Shaffer et al., 2014). It is thus natural to enquire whether blood pressure may be modulated through breathing at this frequency.

In this paper, we ran a study involving participants who followed a 15 min practice of device guided slow, mindful breathing. We found evidence that mindful breathing at a rate of six breaths per minute for 15 min a day is an effective technique in the management of hypertension. In particular, we found the following:

• There was a short term decrease in systolic and diastolic measurements following mindful breathing, and this decrease cannot be attributed to sitting quietly.
• For initial systolic measurements in the range 130–139 mmHg, the systolic pressure decreased by an average of 7.9 mmHg. For initial systolic measurements ≥140 mmHg, the average decrease was 11.7 mmHg.
• For initial diastolic measurements in the range 80–90 mmHg, the diastolic pressure decreased by an average of 3.4 mmHg. For initial diastolic measurements ≥90 mmHg, the average decrease was 5.4 mmHg.
• Measured over many days, there is a decrease in resting systolic and diastolic blood pressure. The resting systolic decreased by an average of 4.3 mmHg and the control set showed no decrease. The resting diastolic decreased by an average of 1.6 mmHg after accounting for the decrease seen in the control set.

One of the main outcomes of this analysis is the possibility of a reduction in systolic pressure, arising from a daily practice of mindful breathing for 15 min. We found that on average, the systolic pressure decreased by 4.3 mmHg, while the control set showed no reduction in systolic pressure. The time to reach 90% of the asymptotic value was found to be 15.2 days. We also found strong evidence for a dose-dependent effect for small values of d (see Eq. 2). The diastolic pressure shows only a small reduction of 1.6 mmHg when accounting for the decrease seen in the Control set. Another significant finding was that the reduction in systolic pressure depends on hypertension status, and medication usage, but not age or sex. Non-pharmacological treatments such as device guided mindful breathing were found to be beneficial to participants with hypertension, and this technique may contribute to overall care in the management of hypertension. Although we have only considered slow breathing in this work, movement exercises coupled with breathing, e.g., Tai Chi have also shown promise in the non-pharmacological management of hypertension (Campo et al., 2015; Wen and Su, 2021). Campo et al. (2015) conducted a 12-week randomized control trial involving 63 female senior cancer survivors and found that the Tai Chi group had significantly lower systolic blood pressure with no statistically significant difference in diastolic pressure. Wen and Su (2021) conducted a 6-week randomized control trial to study the health benefits of simplified and Wu-style Tai Chi on the middle aged and elderly. They found that both Tai Chi groups showed a significant decrease in systolic pressure compared to the baseline, while only the Wu-style Tai chi group showed a decrease in diastolic pressure

The Framingham Risk Score (D Agostino et al., 2008) is a useful technique to quantify the risk of cardiovascular disease. Elevated systolic pressure is one of the biggest modifiable risk factors for adverse cardiovascular events (the hazard ratio for the logarithm of systolic pressure when not treated with medications is 15.82 for females, and 6.91 for males) (D Agostino et al., 2008). The formula assigns a score indicative of the 10-year risk of developing cardiovascular disease, taking into account the person’s age, sex, systolic blood pressure, total cholesterol, high density lipoprotein (HDL) cholesterol, smoking status, diabetes status, and whether blood pressure is controlled using medications. Due to the high hazard ratio for systolic pressure, even a modest reduction in systolic pressure can lead to a significant reduction in the risk of cardiovascular disease. As an example, consider an individual of age 40, with a total cholesterol of 200 mg/dL, HDL of 50 mg/dL, and no history of smoking, diabetes, or blood pressure medications. Reducing the systolic blood pressure by 4.3 mmHg can reduce the 10 years risk of adverse cardiovascular events by as much as 9.4% (8.7%) for females with an initial systolic of 120 (130) mmHg. For males, the equivalent decrease in risk is 6.6% (6.0%) for an initial systolic of 120 (130) mmHg.

There are several limitations to our work. A significant limitation is that blood pressure values were self reported, i.e., there were no medical personnel to collect the data. Blood pressure recordings were taken from single measurements, i.e., one measurement prior to mindful breathing, and one measurement after mindful breathing. Although this practice is consumer friendly, it is less precise than computing the mean of a number of measurements. Selection bias may be present, as participants were selected from individuals who own a Fitbit device and an FDA or Health Canada approved blood pressure measuring device. The control group is slightly older and skews male, although we do not find age or gender to be confounders. A follow-up study with more control participants may strengthen these findings. The study duration of 28 days did not permit us to do long term assessments of blood pressure. Although these various limitations cannot be ignored, this study highlights the major role that commercial wearable devices can play in the management of hypertension.

## Data Availability

The original contributions presented in the study are included in the article/Supplementary Material, further inquiries can be directed to the corresponding author.

## Footnotes

1
https://www.resperate.com/
