# Supplementary material for: Mindful breathing as an effective technique in the management of hypertension
Source: Front Physiol. 2024 Jan 23;14:1339873. doi: 10.3389/fphys.2023.1339873 (PMC10844494; doi:10.3389/fphys.2023.1339873)
Supplement: Supplementary file 1 [file DataSheet1.DOCX]

Supplementary Material

1. **The effect of steps on blood pressure**

It is commonly accepted that blood pressure measurements should only be taken after the individual has been seated for several minutes. In the text, we imposed the condition that participants wore their Fitbit device for five minutes prior to mindful breathing, and took fewer than 10 steps in that five minute period. When this is not enforced, the decrease in blood pressure post mindful breathing will be higher because movement may have increased the blood pressure prior to starting the practice. In this section, we investigate this effect in more detail.

Figure S1 shows the effect of movement on blood pressure. Figure S1 shows the reduction in systolic (blue, (a) and (c)) and diastolic (red, (b) and (d)) blood pressure, for initial systolic measurements >= 130 mmHg or diastolic measurements >= 80 mmHg. Δ_systolic_ and Δ_diastolic_ show the change (after – before) in systolic and diastolic pressure following mindful breathing. For plots (a) and (b), the horizontal axis shows the *maximum* number of steps taken in the 5 minute period prior to starting the mindful breathing practice. When we restrict individuals to only those who take fewer than 10 steps in the 5 minutes prior to mindful breathing, the average decrease in systolic (diastolic) pressure is 9.7 (3.9) mmHg (considering only individuals with initial systolic >= 130 mmHg or diastolic >= 80 mmHg). When there is no restriction on movement, the decrease in blood pressure can appear to be as high as 10.9 (4.1) mmHg for the systolic (diastolic) measurements. In plots (c) and (d), the horizontal axis represents the *minimum* number of steps taken in the 5 minutes prior to mindful breathing, i.e. we consider individuals who took at least a certain number of steps prior to starting the practice. When we restrict participants to those who took more than 100 steps prior to mindful breathing, the average decrease in systolic (diastolic) was 15.1 (5.0) mmHg (again, only considering individuals with initial systolic >= 130 mmHg or diastolic >= 80 mmHg). This reduction in blood pressure is 5.4 (1.1) mmHg in excess of the true reduction due to mindful breathing, and the excess may be attributed to movement. It is thus very important to restrict movement prior to taking a blood pressure measurement. We also note that the impact of movement is higher for systolic pressure.


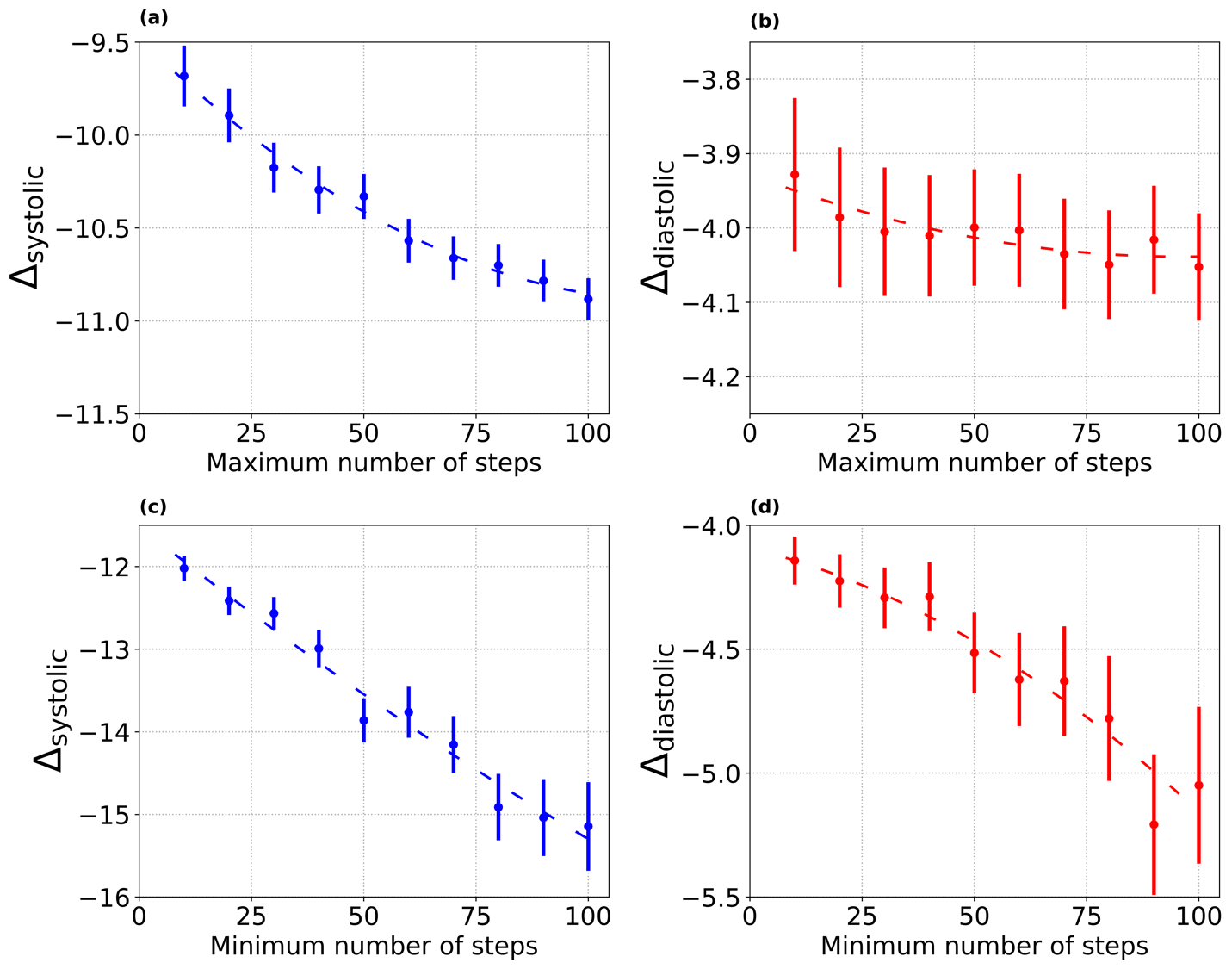


*Figure S1: Effect of movement on blood pressure. Plots (a) and (b) show blood pressure reduction following mindful breathing considering individuals with* ***at most*** *x number of steps in the 5-minute period prior to the practice. Plots (c) and (d) consider individuals with* ***at least*** *x number of steps.*

1. **Robustness to mean reversion**

The values of 𝛼_0_ for the changes in systolic (97.0 mmHg) and diastolic (63.4 mmHg) blood pressure are much lower than the mean of the distribution for systolic blood pressure (125.3 mmHg) and diastolic blood pressure (76.5 mmHg) prior to the mindful breathing intervention. If an individual’s blood pressure measurement may be modeled by a normal distribution with a mean and standard deviation, then successive measurements will in general, result in a difference in readings. If the first reading is higher than the mean, there is a greater than random chance for the second reading to be lower than the mean, resulting in a negative shift, and potentially mimicking our results. This is the well known mean reversion effect. However, the difference between readings is as likely to be negative as it is to be positive. Therefore if the blood pressure measurements for a given individual are drawn from a normal distribution and the observed effect is due to mean reversion alone, we would expect 𝛼_0_ to be close to the mean of the systolic and diastolic distributions. Since the observed 𝛼_0_ is far from the mean, the decrease in blood pressure cannot be satisfactorily explained by mean reversion. The strong statistical significance for the effect size (*d* = 0.3925 for systolic, and *d* = 0. 2144 for diastolic) also offers further evidence that the results cannot be explained by mean reversion alone (which would predict *d* = 0). Thus, while we cannot exclude the contribution due to mean reversion, we do not believe that mean reversion can suitably explain the difference in blood pressure measurements.
